# TGF-β mediates early angiogenesis and latent fibrosis in an Emilin1-deficient mouse model of aortic valve disease

**DOI:** 10.1242/dmm.015255

**Published:** 2014-08

**Authors:** Charu Munjal, Amy M. Opoka, Hanna Osinska, Jeanne F. James, Giorgio M. Bressan, Robert B. Hinton

**Affiliations:** 1 Division of Cardiology, The Heart Institute, Cincinnati Children’s Hospital Medical Center, Cincinnati, OH, USA; 2 Division of Molecular Cardiovascular Biology, The Heart Institute, Cincinnati Children’s Hospital Medical Center, Cincinnati, OH, USA; 3 Department of Molecular Medicine, University of Padua, 35121 Padua, Italy

**Keywords:** Elastic fibers, Extracellular matrix, Aortic valve, Fibrosis, Angiogenesis

## Abstract

Aortic valve disease (AVD) is characterized by elastic fiber fragmentation (EFF), fibrosis and aberrant angiogenesis. Emilin1 is an elastin-binding glycoprotein that regulates elastogenesis and inhibits TGF-β signaling, but the role of *Emilin1* in valve tissue is unknown. We tested the hypothesis that *Emilin1* deficiency results in AVD, mediated by non-canonical (MAPK/phosphorylated Erk1 and Erk2) TGF-β dysregulation. Using histology, immunohistochemistry, electron microscopy, quantitative gene expression analysis, immunoblotting and echocardiography, we examined the effects of *Emilin1* deficiency (*Emilin1*^−/−^) in mouse aortic valve tissue. *Emilin1* deficiency results in early postnatal cell-matrix defects in aortic valve tissue, including EFF, that progress to latent AVD and premature death. The *Emilin1*^−/−^ aortic valve displays early aberrant provisional angiogenesis and late neovascularization. In addition, *Emilin1*^−/−^ aortic valves are characterized by early valve interstitial cell activation and proliferation and late myofibroblast-like cell activation and fibrosis. Interestingly, canonical TGF-β signaling (phosphorylated Smad2 and Smad3) is upregulated constitutively from birth to senescence, whereas non-canonical TGF-β signaling (phosphorylated Erk1 and Erk2) progressively increases over time. *Emilin1* deficiency recapitulates human fibrotic AVD, and advanced disease is mediated by non-canonical (MAPK/phosphorylated Erk1 and Erk2) TGF-β activation. The early manifestation of EFF and aberrant angiogenesis suggests that these processes are crucial intermediate factors involved in disease progression and therefore might provide new therapeutic targets for human AVD.

## INTRODUCTION

Aortic valve disease (AVD) affects more than 2% of the general population and typically manifests later in life (Nkomo et al., 2006). Therapeutic intervention remains primarily surgical valve replacement, which is associated with limited durability and significant complications (Keane et al., 1993; Hammermeister et al., 2000). AVD is characterized by extracellular matrix (ECM) abnormalities that typically manifest as fibrosis then calcification, resulting in stenosis with or without regurgitation. The prevailing view has been that injury and inflammation result in AVD, but there is increasing evidence that the cell-ECM changes that characterize AVD are mediated by abnormalities in molecular programs that regulate cardiac development (Markwald et al., 2010; Hinton and Yutzey, 2011). Our understanding of the molecular mechanisms underlying AVD pathogenesis, especially disease progression, has been limited, in part, by a lack of animal models that recapitulate the natural history of human AVD (Schoen, 2008; Rajamannan et al., 2011).

The mature aortic valve comprises three cusps that are hinged to a crown-shaped annulus within the aortic root. Cusp trilaminar ECM is stratified into fibrosa, spongiosa and ventricularis layers with elastic fibers organized as filaments in the ventricularis layer (Schoen, 1997; Hinton et al., 2006). Valve interstitial cells (VICs) comprise a heterogeneous population of cells that can be classified as quiescent or activated (Rabkin-Aikawa et al., 2004), and some VICs possess characteristics similar to those of smooth muscle cells (SMCs) (Bairati and DeBiasi, 1981; Filip et al., 1986). Activated VICs have been implicated in AVD because they are associated with pathological cell proliferation and maladaptive ECM remodeling (Rabkin-Aikawa et al., 2004). Although healthy adult valve tissue is avascular (Duran and Gunning, 1968), neovascularization has been described in non-rheumatic degenerative AVD and attributed to end-stage inflammatory processes, a secondary result of a wound-healing-like response to injury (Rajamannan et al., 2005; Paruchuri et al., 2006; Li et al., 2011). Little is known about the potential primary role of aberrant angiogenesis in AVD.

Elastic fiber fragmentation (EFF) has long been associated with AVD, and the conventional view is that structural degeneration reflects end-stage disease processes (Schoen, 1997). Mouse models with elastic fiber assembly defects often have valve abnormalities (Hanada et al., 2007; Hinton et al., 2010), and EFF is present in pediatric, as well as adult, AVD (Fondard et al., 2005; Hinton et al., 2006; Wirrig et al., 2011), suggesting that EFF reflects elastic fiber assembly defects, in addition to elastase initiated EFF, and therefore might have a role in AVD progression. EFF has been associated with increased elastolytic enzyme activity, which has been identified as a factor contributing to AVD progression in both mouse and human (Fondard et al., 2005; Krishnamurthy et al., 2012). Elastic fibers are made up of elastin (the core protein) and microfibrils (fibrillins and associated proteins), as well as various glycoproteins, such as emilins and fibulins (Kielty et al., 2002; Wagenseil and Mecham, 2009). Emilin1 (elastin microfibril interface-located protein) is an elastin- and fibulin-5-binding protein that is necessary for elastogenesis and inhibits transforming growth factor β (TGF-β) signaling (Zanetti et al., 2004; Zacchigna et al., 2006). In addition, fibroblasts, keratinocytes and lymphatic endothelial cells in Emilin1-deficient mice demonstrate increased proliferation due to absent Emilin1-integrin interactions (Danussi et al., 2011; Danussi et al., 2012). Importantly, *Emilin1* is expressed in the developing and mature heart valves (Braghetta et al., 2002; Angel et al., 2011; Votteler et al., 2013), and *Emilin1* deficiency results in EFF and TGF-β activation in the aorta. The role of *Emilin1* in AVD pathogenesis is unknown.

**TRANSLATIONAL IMPACT**

**Clinical issue**

Aortic valve disease (AVD) is a major cause of cardiovascular morbidity and affects more than 2% of the general population of the USA. Aortic valve replacement remains the principal treatment strategy for end-stage AVD; however, this process is associated with considerable complications. Presently, there are no pharmacologic therapies to directly treat AVD. A better understanding of the cellular and molecular events that underlie AVD progression is required to develop novel approaches to treatment. Therefore, animal models that mimic the natural history of human AVD are needed to allow researchers to examine the early disease process and test new therapies. *Emilin1* is an essential elastogenic protein that is present in both developing and mature aortic valve tissue. Previous studies have shown that Emilin1 is required for normal elastic fiber assembly and inhibits TGF-β signaling in vasculature. However, the role of Emilin1 dysregulation in AVD pathogenesis is unknown.

**Results**

In this study, the *Emilin1* homozygous knockout mouse (*Emilin1*^−/−^) was identified as a new model of latent AVD. Histological and molecular analyses demonstrated that Emilin1 deficiency is associated with the activation of non-canonical (phosphorylated Erk1 and Erk2) and canonical (phosphorylated Smad2 and Smad3) signaling in aortic valve tissue. This results in early elastase-mediated elastic fiber fragmentation and aberrant angiogenesis in association with early valve interstitial cell (VIC) activation. Interestingly, there is progressive up-regulation of phosphorylated Erk1 and Erk2 signaling over time, which results in the activation of a subset of VICs with myofibroblast-like characteristics and marked progression of valve pathology that shows severe fibrosis, neovascularization and inflammation.

**Implications and future directions**

Together, these results demonstrate that the *Emilin1*-deficient mouse is a unique model of latent fibrotic AVD that provides important insights into early and intermediate disease mechanisms. These findings establish a central role for elastic fiber dysregulation in early AVD pathogenesis, suggesting that faulty elastic fiber assembly and consequent elastase-mediated tissue injury contribute to disease initiation and progression. A better understanding of these early disease processes could contribute to the development of novel treatments for AVD. Because the manifestation of AVD is latent in this model, similar to human AVD, preclinical studies using the Emilin1-deficient mouse model of AVD are warranted.

We tested the hypothesis that *Emilin1* deficiency results in AVD, which is characterized by early EFF and late fibrosis, mediated by non-canonical TGF-β signaling [through mitogen-activated protein kinases (MAPK) and extracellular-signal-regulated kinases 1 and 2 (Erk1/2)]. We have demonstrated here that *Emilin1*^−/−^ aortic valves exhibit EFF and early aberrant angiogenesis, as well as late fibrosis and stenosis, and that these pathologic findings are due to complex TGF-β dysregulation, including progressive upregulation of non-canonical phosphorylated Erk1/2 signaling. This new model of AVD recapitulates the natural history of human AVD, thereby providing insights into mechanisms of disease progression that might identify new therapeutic targets.

## RESULTS

### *Emilin1*^−/−^ aortic valve tissue is characterized by early EFF and progressive elastolysis

**Fig. 1.**
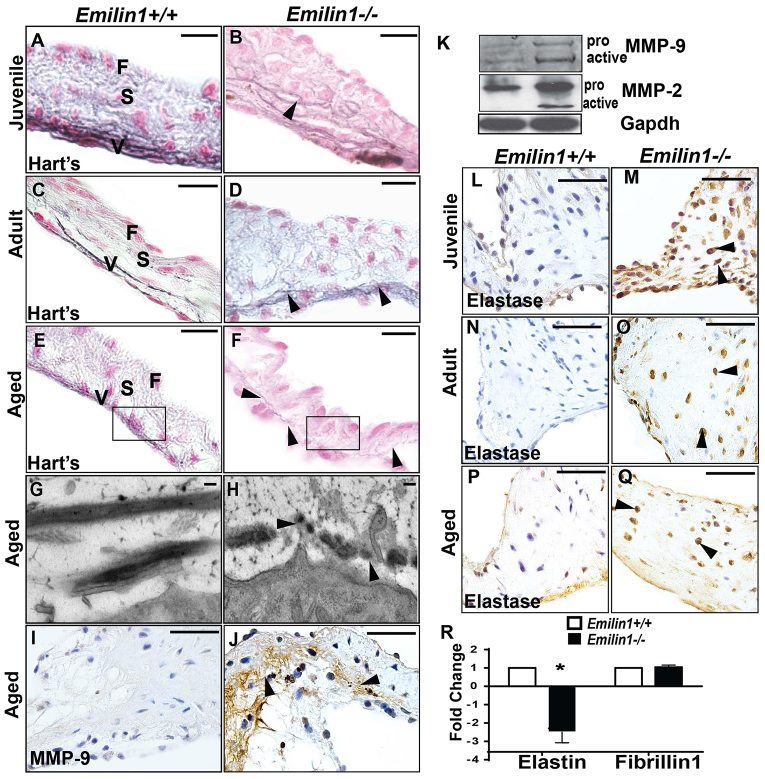
**Elastic fiber assembly defects in *Emilin1*^−/−^ aortic valve tissue.** Sections stained using Hart’s stain show intact elastic fiber filaments localized to the ventricularis layer in *Emilin1*^+/+^ mice (A,C,E) and EFF in *Emilin1*^−/−^ aortic valves that worsened with time (arrowheads, B,D,F). Ultrastructure analyses in aged *Emilin1*^−/−^ aortic valves show EFF with dispersion (arrowheads, H) when compared with intact elastic fiber filaments in *Emilin1*^+/+^ valves (G). MMP-9 and MMP-2 expression increased in the aged aortic valve of *Emilin1*^−/−^ mice when compared with that of *Emilin1*^+/+^ valves using immunohistochemistry (J) and western blotting (K). Western blotting analysis showed increased expression of both the inactive proenzyme form (pro) and the active form of MMP-2 and MMP-9 in *Emilin1*^−/−^ aortic valves (K). There was increased neutrophil elastase expression in *Emilin1*^−/−^ aortic valve tissue, especially at the hinge region, at the juvenile, adult and aged stages (arrows, M,O,Q). In aged *Emilin1*^−/−^ aortic valve tissue, elastin mRNA expression decreased significantly but there was no change in the amount of fibrillin1 mRNA (R). All panels are oriented to show the aortic valve in cross-section with the ventricularis layer situated at the bottom. Mean±s.e.m.; *n*=12; *P<0.05 *Emilin1*^+/+^ versus *Emilin1*^−/−^. Scale bars: 10 μm (A–F); 500 nm (G,H); 50 μm (I–P). F, fibrosa; S, spongiosa; V, ventricularis.

Juvenile *Emilin1*^−/−^ aortic valve tissue exhibits EFF, which is characterized by decreased elastic fiber content and dispersion of elastic fiber components to all cusp layers, namely the fibrosa, spongiosa and ventricularis, as well as the annulus, indicating faulty elastic fiber assembly (Fig. 1A,B). EFF and delamination worsens over time (Fig. 1B,D,F). Ultra-structural analysis of aged *Emilin1*^−/−^ aortic valves confirmed marked EFF, delamination and dispersion (Fig. 1H). MMP-9 was examined because it has been shown to play a crucial role in pathological valve remodeling. MMP-9 expression was unaltered in adult *Emilin1*^−/−^ valves but was increased in aged *Emilin1*-deficient aortic valves in comparison with age-matched controls (Fig. 1J) (Fondard et al., 2005). This observation was supported by immunoblot analyses, which demonstrated increased expression of active MMP-9, as well as MMP-2, in aged mutant valves (Fig. 1K). Importantly, elastase expression, which is not expressed in normal healthy valve, was increased in juvenile and adult *Emilin1*^−/−^ aortic valve tissue and further increased at the aged stage (Fig. 1M,O,Q), demonstrating a role for elastase and remodeling enzymes in the progression of AVD. In aged *Emilin1*^−/−^ aortic valve tissue, elastin mRNA expression was significantly decreased, whereas that of fibrillin-1 was unchanged (Fig. 1R). Elastin mRNA expression was also decreased in ascending aorta and left myocardial tissue that had been isolated from *Emilin1*^−/−^ mice (supplementary material Fig. S2O). Taken together, elastic fiber assembly defects in *Emilin1*^−/−^ mice predispose aortic valve tissue to progressive elastolysis and worsening EFF.

### Aberrant provisional angiogenesis is an early finding that progresses to neovascularization in *Emilin1*^−/−^ aortic valve tissue

**Fig. 2.**
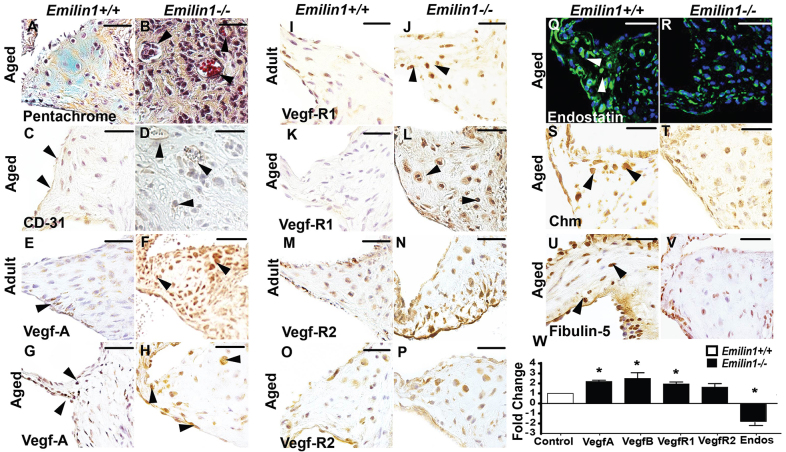
**Emilin1 deficiency is associated with aberrant provisional angiogenesis and neovascularization.** Overt neovascularization is shown in aged *Emilin1*^−/−^ aortic valve tissue, restricted to the annulus and proximal cusp, as indicated by Movat’s pentachrome staining (arrowheads, B) and CD-31-positive stained neovessels (arrowheads, D). *Emilin1*^+/+^ controls are shown in A and C. VEGF-A (E-H) and VEGF-R1 (I–L) expression was increased at adult (F,J) and aged (H,L) stages in the interstitium of *Emilin1*^−/−^ aortic valves when compared with that of control *Emilin1*^+/+^ tissue, which shows normal expression around the endothelial layer (arrowheads, E,G). Levels of endostatin (Q–R, green; blue, nuclei), chondromodulin (Chm) (S–T) and fibulin-5 (U–V) were lower at the aged stage (R,T,V) when compared with *Emilin1*^+/+^ controls (arrowheads, Q,S,U). mRNA expression for VEGF-A, VEGF-R1, VEGF-R2 and endostatin (Endos) in aged *Emilin1*^−/−^ aortic valve tissue relative to *Emilin1*^+/+^ control values (normalized to 1) for each gene (W). Scale bars: 50 μm. Mean±s.e.m.; *n*=10; *P<0.05 *Emilin1*^+/+^ versus *Emilin1*^−/−^.

Neovascularization (overt angiogenesis) was present in *Emilin1*^−/−^ aortic valves at the aged stage only and localized to the annulus and proximal cusp, these neovessels also stained positive for CD-31 (Fig. 2B,D). At the earlier adult stage, *Emilin1*^−/−^ aortic valves exhibited aberrant provisional angiogenesis, characterized by increased interstitial VEGF-A and VEGF-R1 (Flt1) expression, which are normally restricted to the valve endothelium, but unchanged VEGF-R2 (Flk1) expression (Fig. 2F,J,N). In addition, circulatory VEGF-A levels were increased significantly in aged *Emilin1*^−/−^ mice (data not shown). Interestingly, the angiostatic factors chondromodulin (Chm), endostatin and fibulin-5, which are normally ubiquitously expressed in valve tissue, were markedly decreased in aged *Emilin1*^−/−^ aortic valves (Fig. 2R,T,V), suggesting that aberrant angiogenesis is the result of both increased pro-angiogenic factors and decreased angiostatic factors. Quantitative reverse transcription PCR (QRT-PCR) analysis was performed in aged aortic valve tissue that showed a significant increase in both VEGF-A and VEGF-R1, as well as a significant decrease in endostatin. Overall, these results demonstrate that aberrant angiogenesis is an early disease process that worsens over time, identifying a crucial role for angiogenesis in AVD progression.

### Dynamic activation of non-canonical TGF-β (phosphorylated Erk1/2) signaling in *Emilin1*^−/−^ aortic valve tissue

**Fig. 3.**
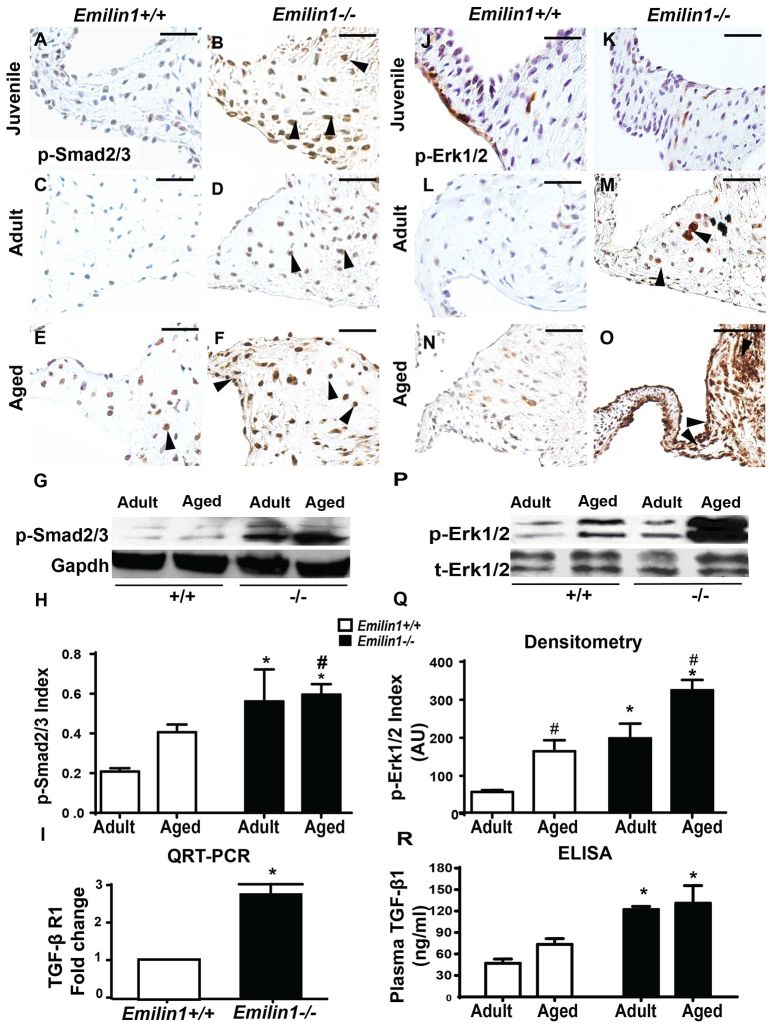
***Emilin1* deficiency is associated with canonical and non-canonical TGF-β activation in aortic valve tissue.** Levels of phosphorylated Smad2/3 (p-Smad2/3) are increased in juvenile (arrowheads, B), adult (arrowheads, D) and aged (arrowheads, F) *Emilin1*^−/−^ aortic valves compared those in juvenile (A), adult (C) and aged (E) *Emilin1*^+/+^ valves. Increased phosphorylated Smad2/3 in adult and aged tissue was confirmed by immunoblotting (G), quantification of Smad2/3-positive nuclei to total nuclei is shown in H. QRT-PCR showed increased mRNA expression of TGF-βR1 in aged *Emilin1*^+/+^ (white bar) and *Emilin1*^−/−^ (black bar) aortic valve (I). MAPK/phosphorylated Erk1/2 signaling progressively increased in *Emilin1*^−/−^ aortic valves (arrowheads, K,M,O) when compared with age-matched controls (J,L,N). Increased phosphorylated Erk1/2 activation (p-Erk1/2) was confirmed by immunoblotting (P) in both adult and aged *Emilin1*^−/−^ aortic valve tissue; this increase was significant at the aged stage, as assessed using ImageJ densitometry quantification (Q). Histogram shows circulatory levels of active TGF-β in the plasma samples of *Emilin1*^+/+^ and *Emilin1*^−/−^ mice (R). Scale bars: 50 μm. Mean±s.e.m.; *n*=14; *P<0.05 *Emilin1*^+/+^ versus *Emilin1*^−/−^; ^#^*P*<0.05 adult versus aged.

To determine the effects of *Emilin1* deficiency on TGF-β signaling in aortic valve tissue, canonical and non-canonical pathways were examined. In *Emilin1*^−/−^ aortic valves at the juvenile stage, levels of phosphorylated Smad2 and Smad3 (Smad2/3) were increased when compared with age-matched controls (Fig 3A,B). In *Emilin1*^−/−^ aortic valves at both the adult and aged stages, there was a significant and similar increase in the absolute number and ratio of phosphorylated Smad2/3-positive nuclei, as well as protein expression using immunoblotting (Fig. 3D,F–H). In *Emilin1*^+/+^ aortic valves, phosphorylated Smad2/3 was increased with advanced age but significantly decreased when compared with that of *Emilin1*^−/−^ valves. There was no change in phosphorylated Smad2/3 in *Emilin1*^−/−^ myocardium (supplementary material Fig. S2G). TGF-βR1 mRNA expression was increased in *Emilin1*^−/−^ aortic valve tissue, as determined by using QRT-PCR (Fig. 3I). The amount of phosphorylated Erk1/2 was unchanged in juvenile *Emilin1*^−/−^ aortic valves but was modestly increased in adult *Emilin1*^−/−^ aortic valves (Fig. 3J–M) and dramatically increased at the aged stage when compared with the younger mutant (Fig. 3M,O), identifying progressive phosphorylated Erk1/2 activation. There was no phosphorylated Erk1/2 expression in *Emilin1*^−/−^ myocardium (data not shown). However, aged *Emilin1*^−/−^ aorta tissue demonstrated increased phosphorylated Erk1/2 expression that was localized to the aortic root (supplementary material Fig S2I,N). Circulatory plasma levels of active TGF-β were significantly increased at the adult and aged stages in *Emilin1*-deficient mice (Fig. 3R). Taken together, these findings demonstrate dynamic dysregulation of TGF-β signaling in *Emilin1*^−/−^ aortic valve tissue, indicating a possible role for non-canonical phosphorylated Erk1/2 activation in AVD progression.

### Early VIC activation and proliferation is maladaptive in *Emilin1*^−/−^ aortic valve tissue

**Fig. 4.**
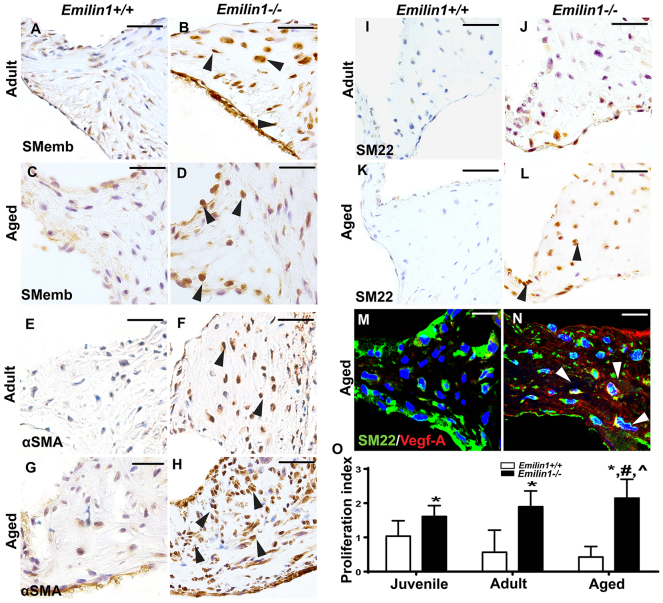
***Emilin1* deficiency is associated with early VIC and late myofibroblast activation in aortic valve tissue.** VIC activation is demonstrated by increased SMemb and αSMA expression at the adult (arrowheads, B,F) and aged stages (arrowheads, D,H) in *Emilin1*^−/−^aortic valves when compared with *Emilin1*^+/+^ valves (A,C,E,G). Myofibroblast activation is shown by increased SM22 at the aged stage (arrowheads, L) but not the adult stage (J) in *Emilin1*^−/−^ aortic valves when compared with *Emilin1*^+/+^ valves (I,K). Confocal imaging shows colocalization of SM22 (green) and VEGF-A (red) in the endothelium of *Emilin1*^+/+^ (M) and the interstitium of *Emilin1*^−/−^ aortic valve tissue (white arrowheads, N). *Emilin1*^−/−^ aortic valves demonstrate an increase in the proliferation index at all stages when compared with age-matched *Emilin1*^+/+^ valves (O). Aged *Emilin1*^−/−^ aortic valves also showed significantly increased proliferation when compared with juvenile and adult *Emilin1*^−/−^ aortic valves. Scale bars: 50 μm (A-L); 10 μm (M,N). Mean±s.e.m.; n=8; *P<0.05 *Emilin1*^+/+^ vs *Emilin1*^−/–; #^*P*<0.05 adult *Emilin1*^−/−^ vs aged *Emilin1*^−/–; ^^*P*<0.05 juvenile *Emilin1*^−/−^ vs aged *Emilin1*^−/−^.

In order to examine the VIC phenotype, embryonic smooth muscle myosin heavy chain (SMemb) and alpha smooth muscle actin (αSMA) were studied in *Emilin1*^−/−^ aortic valve tissue. SMemb expression was increased at both the adult and aged stages, indicating VIC activation (Fig. 4B,D). αSMA expression was similarly increased at the adult stage but dramatically increased at the aged stage (Fig. 4F,H). SM22, which is a myofibroblast marker, was not expressed in *Emilin1*^−/−^ aortic valve tissue at the adult stage but was abundantly expressed at the aged stage (Fig. 4J,L), identifying a distinct cell activation pattern consistent with latent myofibroblast-like cell activation. Interestingly, VEGF-A and SM22 were colocalized in some VICs (Fig. 4N), primarily in the annulus region and proximal cusp where neovessels are present, demonstrating that a subset of VICs, rather than endothelial cells, regulate VEGF-A production in the valve interstitium, consistent with previous observations (Paranya et al., 2001; Paruchuri et al., 2006; Syväranta et al., 2010). The total number of proliferative cells was quantified by proliferation index analysis using phosphorylated histone H3 (HH3) staining. The results showed an increased proliferative index in *Emilin1*^−/−^ aortic valve tissue at the juvenile and adult stages that significantly increased by the aged stage (Fig. 4O). Ki67 staining demonstrated similar patterns of proliferation by stage and genotype (supplementary material Fig. S1O). Importantly, activated VICs were also positive for the proliferation marker phosphorylated HH3, indicated by colocalization of phosphorylated HH3 and SMemb (supplementary material Fig. S1L). Overall, these results implicate early VIC activation and late myofibroblast-like cell activation in the latent manifestation of AVD.

### The aged *Emilin1*^−/−^ aortic valve demonstrates fibrosis and inflammation, but no calcification

**Fig. 5.**
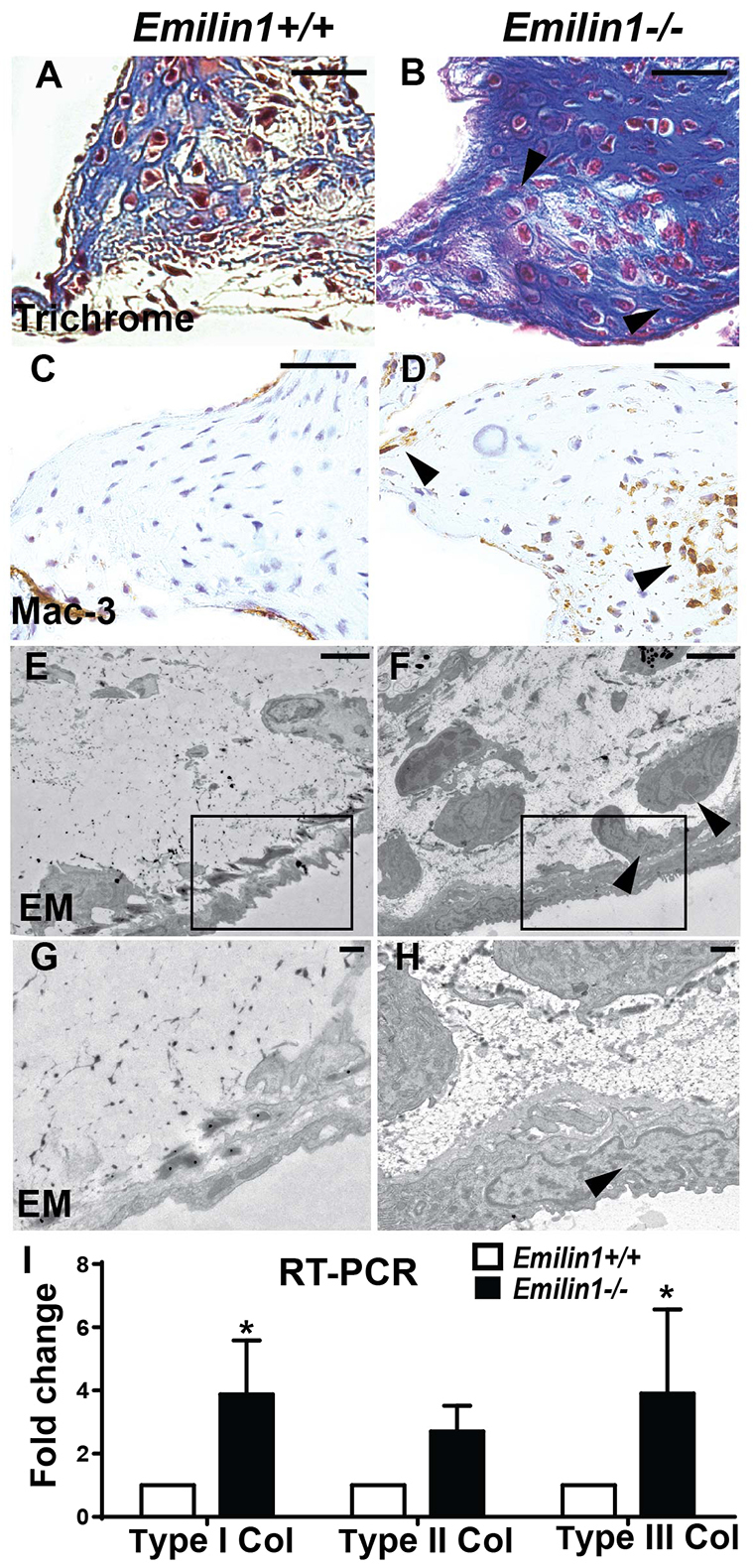
**Aged *Emilin1*^−/−^ aortic valve tissue demonstrates fibrosis and inflammation.** Increased collagen deposition (blue staining using Masson’s trichrome) is seen in the *Emilin1*^−/−^ aortic valve annulus (arrowheads, B) when compared with age-matched *Emilin1*^+/+^ valves (A). Macrophage infiltration, as evidenced by Mac-3-positive cells (arrowheads, D), is present in aged *Emilin1*^−/−^ valves only and not *Emilin1*^+/+^ controls (C). Electron microscopy (EM) of *Emilin1*^−/−^ aortic valves showed fibroblast-like cells in all cusp layers (arrowheads, F,H) along with collagen accumulation. Relative mRNA expression of type I, II and III collagen in the aortic valve tissue of aged *Emilin1*^−/−^ mice to that of *Emilin1*^+/+^ mice (I). Scale bars: 50 μm (A–D); 500 nm (E,F); 100 nm (G,H). Mean±s.e.m.; *n*=14; *P<0.05 *Emilin1*^+/+^ versus *Emilin1*^−/−^.

Fibrillar collagen deposition in aortic valve tissue was increased in aged *Emilin1*^−/−^ when compared with *Emilin1*^+/+^. Specifically, type I and type III collagen were increased significantly (Fig. 5G). There was evidence of mild aortic valve fibrosis at the earlier adult stage (data not shown). To determine the potential role of inflammation in the manifestation of AVD in *Emilin1*-deficient mice, we examined macrophage and leukocyte markers. *Emilin1*^−/−^ aortic valves displayed abundant macrophage expression (Mac-3; also known as Lamp2) at the aged stage (Fig. 5D), but not earlier stages (data not shown). Interestingly, CD-45, a pan-leukocyte marker, remained unchanged compared with controls at all stages (data not shown). Ultrastructure analysis showed a qualitative abundance of fibroblast-like cells in the hinge region of aged *Emilin1*^−/−^ aortic valves (Fig. 5F,H). The identity of fibroblast-like cells was further established by the presence of vimentin, an intermediate cytoskeletal protein present in myofibroblast cells, which was increased in *Emilin1*^−/−^ aortic valve tissue (supplementary material Fig. S1B). Interestingly, *Emilin1*-deficient aortic valves did not exhibit calcification at any stage, as shown by the calcification marker Alizarin Red and Runx-2 (supplementary material Fig. S1F,H). Overall, these findings identify a role for *Emilin1* deficiency in the latent development of valve fibrosis, similar to the natural history of human AVD.

Latent fibrosis was also present in the ascending aorta and myocardium of *Emilin1*^−/−^ mice, as evidenced by ultrastructural and morphometric analysis (supplementary material Fig. S2). Excessive collagen deposition was largely restricted to the perivasculature regions of the myocardium and adventitial region of the ascending aorta (supplementary material Fig. S2D,K). *Emilin1*^−/−^ myocardium showed altered sarcomere morphology that was characterized by an increased cisternal space due to gaps between the sarcomeres and sarcoplasmic reticulum, and contracted sarcomeres with thinner Z-discs (supplementary material Fig. S2F); in addition, mitochondrial number and morphology were unaltered, consistent with a nonspecific Ca^2+^ handling abnormality. Periostin, a profibrotic marker, was increased in the left ventricular myocardium of aged *Emilin1*^−/−^ (supplementary material Fig. S2G). Collagen fibers in the media of *Emilin1*^−/−^ aorta were randomly arranged and heterogeneous in size (supplementary material Fig. S2M). Type I, II and III collagen were not significantly changed in ascending aorta or myocardium, suggesting that other collagens contribute to the fibrotic response in these tissues. Aged *Emilin1*^−/−^ mice also demonstrated thoracic aortic aneurysm or aortopathy, restricted to the aortic root region, and three out of 10 (30%) mice demonstrated aortic dissection. Overall, these findings identify a potential role for *Emilin1* in regulating ECM in cardiovascular tissues.

### *Emilin1* deficiency results in AVD *in vivo*

In order to evaluate aortic valve function in the *Emilin1*^−/−^ mouse *in vivo*, we performed echocardiography in adult and aged mice. The aortic valve peak velocity and corresponding pressure gradient were significantly increased in aged *Emilin1*^−/−^ mice compared with age-matched controls (Table 1), consistent with aortic valve stenosis. In addition, 20% demonstrated aortic insufficiency. The aortic root index was increased in aged *Emilin1*^−/−^ mice, consistent with mild aortic root dilation, a finding that is commonly associated with AVD (Hahn et al., 1992). The ascending aorta dimension was normal. Paradoxically, the aortic valve annulus dimension was increased, which together with aortic root dilation is reminiscent of annulo-aortic ectasia in human connective tissue disorders. In 75% of aged *Emilin1*^−/−^ mice, there was significant left ventricular dysfunction, a common complication of severe AVD. Left ventricular mass and end diastolic dimension were unchanged in *Emilin1*^−/−^ mice, consistent with the absence of a primary cardiomyopathy. Interestingly, 25% of *Emilin1*^−/−^ mice die prematurely between 14 and 18 months due to unclear causes when compared with *Emilin1*^+/+^ mice, which are reported to have a longevity of 25 months (Yuan et al., 2009). No significant changes in aortic valve and ventricular functions were observed in adult mutant mice when compared with age-matched control (data not shown). Taken together, these findings identify the *Emilin1*^−/−^ mouse as a model of latent fibrotic AVD.

**Table 1.**
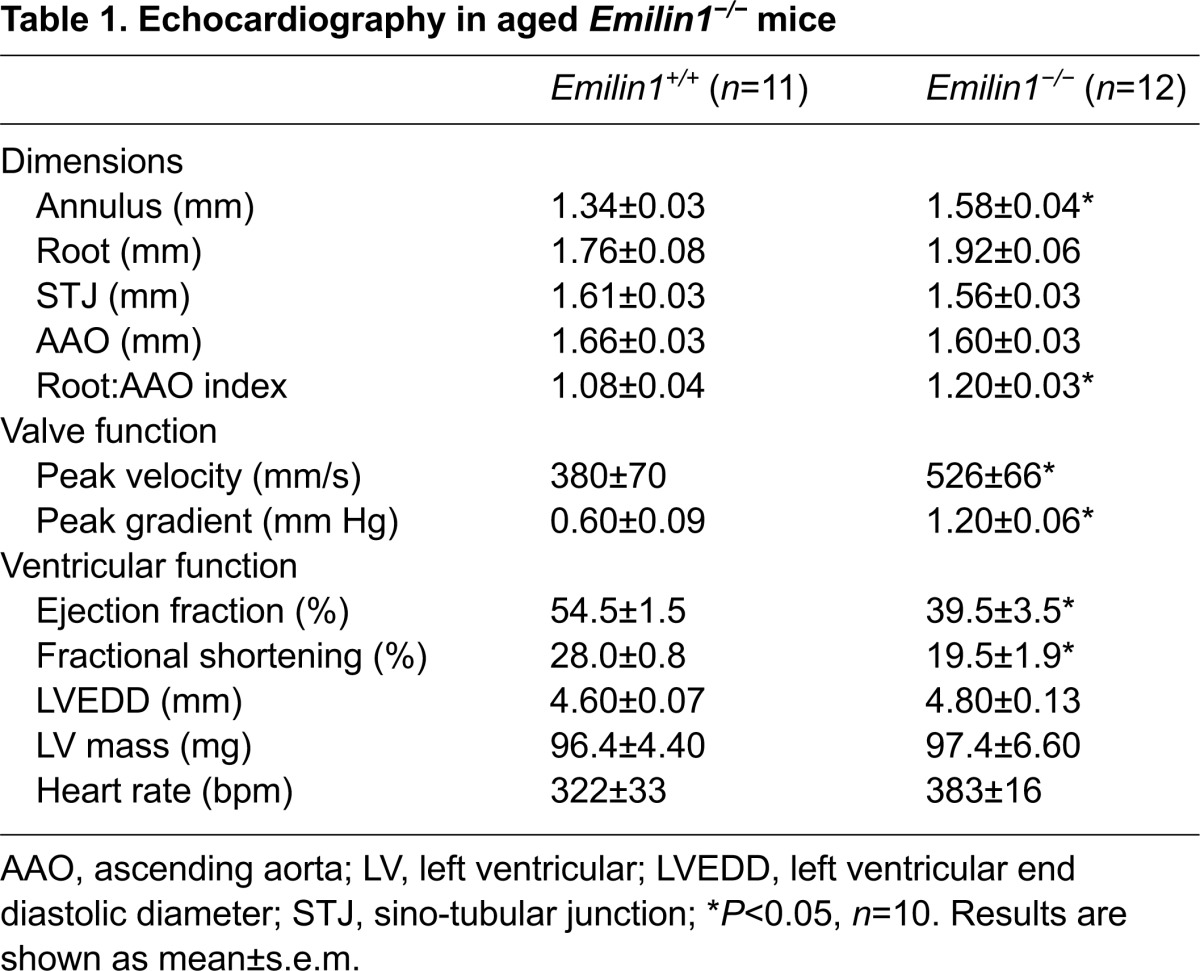
Echocardiography in aged *Emilin1*^−/−^ mice

## DISCUSSION

**Fig. 6.**
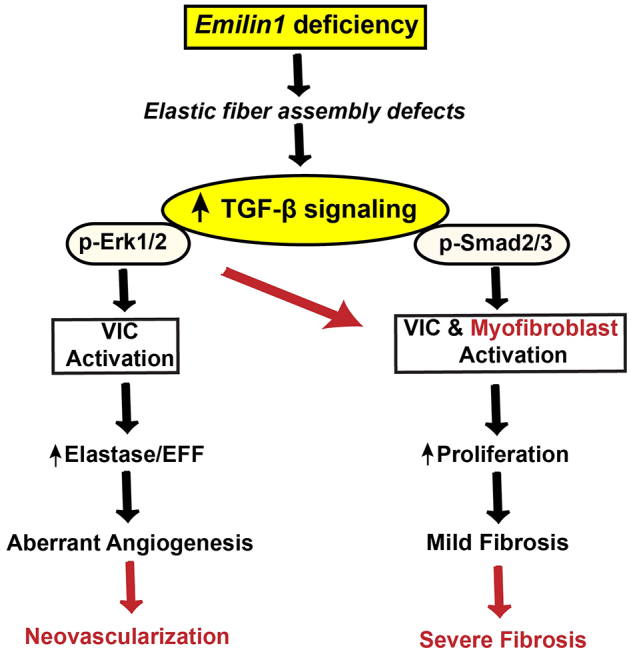
**Hypothetical model of complex TGF-β signaling dysregulation in *Emilin1*-deficient aortic valve tissue.** Emilin1 deficiency results in inherent elastic fiber assembly defects and activation of TGF-β signaling. This results in downstream activation of both non-canonical (phosphorylated Erk1/2; p-Erk1/2) and canonical (phosphorylated Smad2/3; p-Smad2/3) signaling in *Emilin1*^−/−^ aortic valve tissue, resulting in early mild fibrosis and aberrant angiogenesis due to early VIC activation and elastic fiber fragmentation (EFF) mediated, in-part, by increased expression of proteolytic enzymes, such as elastases, MMP-2 and MMP-9. Over time (red), there is a progressive upregulation of phosphorylated Erk1/2 signaling, which results in myofibroblast activation, neovascularization and severe fibrosis at the aged stage.

In the present study, we demonstrated that the *Emilin1* deficient mouse is a model of human fibrotic AVD. Importantly this model recapitulates the natural history of human AVD allowing investigation of early disease processes. In summary, *Emilin1*^−/−^ aortic valve tissue demonstrates complex TGF-β dysregulation that causes downstream activation of both non-canonical (phosphorylated Erk1/2) and canonical (phosphorylated Smad2/3) TGF-β signaling, and ultimately results in aberrant angiogenesis and mild fibrosis in the adult stage due to VIC activation and EFF mediated in-part due to increased expression of elastolytic enzymes. Interestingly, over time, there is progressive upregulation of phosphorylated Erk1/2 signaling that results in activation of a subset of VICs with myofibroblast-like characteristics, which results in neovascularization and severe fibrosis at the aged stage (Fig. 6). Taken together, the *Emilin1*^−/−^ model of AVD provides unique opportunities to identify predictive biomarkers and to test new therapeutic targets that could represent much needed early intervention strategies to prevent advanced AVD and the need for surgery.

Elastic fibers play a crucial role in continuous valve motion by contributing to the normal recoil process during the cardiac cycle, and EFF and elastase-mediated disease progression are universal findings of AVD that contribute to valve dysfunction (Vesely, 1997; Fondard et al., 2005; Schoen, 2008). *Emilin1* deficiency and the resulting elastic fiber assembly defects have been studied in tissue of the aorta, showing that *Emilin1* inhibits TGF-β signaling and that *Emilin1* deficiency causes TGF-β upregulation (Zanetti et al., 2004; Zacchigna et al., 2006). The findings of the current study show that *Emilin1*^−/−^ aortic valves have intrinsic elastic fiber assembly defects, suggesting that EFF might be the result of elastic fiber assembly defects that trigger further elastase-mediated EFF and a progressive disease process. Several mouse models of mutated elastic fiber components have valve defects (Ng et al., 2004; Hanada et al., 2007; Hinton et al., 2010; Krishnamurthy et al., 2012), suggesting that elastic fiber assembly defects have a central role in AVD pathogenesis. Interestingly, in normal aortic valve tissue, *Emilin1* is present in the annulus and other cusp layers, in addition to its presence in the mature elastic fiber filaments in the ventricularis layer, suggesting that *Emilin1* has functional roles additional to those associated with elastic fiber assembly (Angel et al., 2011; Votteler et al., 2013). Overall, this study identifies *Emilin1* as an ECM protein that is necessary for mature valve structure and function, and the *Emilin1*^−/−^ mouse as an important model of viable AVD.

Neovascularization is present in the proximal cusp and annulus regions of aged *Emilin1*^−/−^ aortic valves in proximity to EFF, consistent with findings in human AVD and the idea that cell-ECM homeostasis is disrupted primarily at the hinge region of the valve (Thubrikar et al., 1986; Hinton et al., 2010; Wirrig et al., 2011). In the present study, we have shown a marked imbalance between pro-angiogenic and anti-angiogenic factors that promotes aberrant angiogenesis in the typically avascular valve tissue. Angiogenic signaling pathways regulate normal embryonic valve development (Combs and Yutzey, 2009), but it is largely unknown whether these pathways contribute to the maintenance of mature adult valve tissue. Previous studies have shown that mechanisms of AVD recapitulate developmental programs, suggesting that the dysregulation of specific structural proteins and signaling pathways incite disease and contribute to faulty homeostasis over time and ultimately valve dysfunction later in life (Markwald et al., 2010; Hinton and Yutzey, 2011; Mahler and Butcher, 2011). Elastic fiber fragments, or degradation products, might have pro-angiogenic, pro-proliferative, pro-calcific or pro-inflammatory properties (Perrotta et al., 2011; Pivetta et al., 2014; Parks and Mecham, 2011), in addition to well-described increases in elastase activity (Robinet et al., 2005), suggesting that different elastic fiber fragments have different maladaptive effects. In this context, *Emilin1*-deficiency-related EFF results in aberrant angiogenesis and fibrosis, but not calcification. The identification of specific mechanisms that regulate different types of disease progression might provide new potential therapeutic targets that prevent AVD progression.

The role of canonical TGF-β signaling (phosphorylated Smad2/3) during valve development and disease has been well established (Camenisch et al., 2002; Walker et al., 2004), but little is known about the role of non-canonical TGF-β signaling (phosphorylated Erk1/2). Previous *in vitro* studies have shown that TGF-β1 signaling activates canonical Smad2/3 and non-canonical MAPK pathways, resulting in VIC activation and fibrosis, suggesting a potential role for phosphorylated Erk1/2 in AVD (Gu and Masters, 2009; Hutcheson et al., 2012). Interestingly, we showed that non-canonical signaling was upregulated and progressively increased over time, indicating the presence of a liability threshold for myofibroblast activation (Fig. 6). We showed that VIC activation occurs early in the disease process and that myofibroblast-like cell activation occurred later in the disease process, associated temporally with the latent findings of marked fibrosis, proliferation and inflammation. Activated myofibroblasts release angiogenic factors in the valve interstitium, which is typically avascular, providing a putative mechanism for the advancement of late neovascularization from early provisional aberrant angiogenesis (Paruchuri et al., 2006). Consistently, our findings showed colocalization of activated myofibroblasts with VEGF-A-positive cells. In addition, there was a progressive increase in the proliferation index in *Emilin1*^−/−^ aortic valve tissue, suggesting discrete contributions from each type of VIC activation. Constitutive phosphorylated Smad2/3 upregulation might result in early VIC activation, increased elastolytic activity, proliferation and provisional angiogenesis, whereas progressive phosphorylated Erk1/2 upregulation (or a cumulative threshold of both canonical and non-canonical TGF-β signaling) might result in late myofibroblast activation that results in fibrosis and inflammation, consistent with previous reports (Hutcheson et al., 2013). Previous reports have shown robust activation of phosphorylated Erk1/2 and proliferation in *Emilin1*^−/−^ fibroblast cells that is mediated through a PTEN-dependent pathway (Danussi et al., 2011; Danussi et al., 2012). However, in the current study, PTEN levels were unaltered despite Erk1/2 activation and increased proliferation in *Emilin1*^−/−^ valves, consistent with a role for activated non-canonical TGF-β signaling in aortic valve tissue. A limited number of apoptotic cells were identified as being restricted to the hinge region of aged *Emilin1*^−/−^ aortic valves (supplementary material Fig. S1M), possibly due to increased phosphorylated Erk1/2 signaling and high mechanical strain, suggesting that the biomechanical properties of the *Emilin1*-deficient valves warrant further investigation. This implicates non-canonical MAPK/phosphorylated Erk1/2 activation as a mechanism underlying the progression of AVD and the manifestation of advanced disease, controlling for the important adverse effects of aging.

Although non-resident cells have been described in the valve interstitium of wild-type mice (Hajdu et al., 2011), the inflammation observed in aged *Emilin1*^−/−^ aortic valves might be due to other processes. For example, inflammation might be the direct result of systemic TGF-β upregulation, given the role of TGF-β signaling in inflammation. Alternatively, in light of the abnormalities appreciated in the adventitia layer of the aorta, inflammation might be the result of the migration of non-resident cells from the adventitia layer into the valve annulus and proximal cusp, consistent with the localization of neovascularization. Another alternative is direct infiltration due to a discontinuous endothelial layer. This results in elastases in the valve interstitium and migration of circulating hematopoietic (or non-resident) cells from either the newly formed valve neovasculature, which is not present until the aged stage, or the disrupted endothelium. There were areas of endothelial disruption around the edges of the *Emilin1*^−/−^ aortic valve (data not shown), which is consistent with previous findings (Zanetti et al., 2004; Danussi et al., 2012). Previous evidence suggests that activation of the phosphorylated Erk1/2 pathway is a crucial modifier in elastase-mediated diseases (Preston et al., 2002; Ghosh et al., 2012). Taken together, complex TGF-β dysregulation mediates early and late pathologic findings in *Emilin1*^−/−^ aortic valves, and phosphorylated Erk1/2 activation might play a more important role than previously appreciated in AVD pathogenesis.

The findings of the current study identify the *Emilin1*^−/−^ mouse as a model of latent fibrotic AVD. These findings have substantial clinical implications that emphasize the importance of translational research. AVD progression is a poorly understood process that needs to be defined on a molecular basis in order to test new clinical therapies (Rajamannan et al., 2011). Presently, there are no pharmacological treatment strategies for early-stage AVD; therefore, the elucidation of the natural history of the disease will facilitate preclinical studies aiming to test early intervention strategies, including pharmacological therapies attempting to prevent advanced AVD, potentially encompassing elastase, phosphorylated Erk1/2 or angiogenesis inhibitors. In addition, this model might provide insights into common processes underlying aortopathy associated with AVD. This clinical association is well described and there is some genetic overlap that establishes shared etiologic factors, including TGF-β dysregulation (Hinton, 2012), but this is largely undefined. Mutations in human *EMILIN1* have been associated with cardiovascular disease (Shen et al., 2009; Liu and Xi, 2012), but not specifically AVD or aortopathy, and therefore could represent an important candidate gene and/or genetic modifier. Overall, the emilin family of glycoproteins might represent important factors in cardiovascular diseases.

## MATERIALS AND METHODS

### Animals

*Emilin1* deficient (*Emilin1*^−/−^) and wild-type (*Emilin1*^+/+^) littermates were studied at juvenile (8–10 days), adult (4–6 months) and aged (12–14 months) stages. Mice were maintained on a C57Bl6 genetic background, and genotyping was performed as described previously (Zanetti et al., 2004). All protocols were approved by the Institutional Animal Care and Use Committee at Cincinnati Children’s Hospital Medical Center.

### Histology and immunohistochemistry

Hearts were isolated and processed as described previously (Hinton et al., 2010). Movat’s pentachrome, Masson trichrome, Hart’s and Alizarin-Red stains were used. Immunohistochemistry was performed to assess markers of VIC activation, myofibroblast differentiation, angiogenesis, TGF-β signaling, elastolysis and proliferation as described in Table 2. Antibodies were obtained from Cell Signaling (Boston, MA, USA); Abcam (Cambridge, MA, USA); Santa Cruz Biotechnology (Dallas, TX, USA); Sigma-Aldrich (St Louis, MO, USA); Invitrogen (Grand Island, NY, USA) and Millipore (Billerica, MA, USA) with catalog information and other details given in Table 2. A universal streptavidin-biotin and diaminobenzidine detection system (Pierce) was used for colorimetric detection. Secondary antibodies used for immunofluorescence were goat against mouse IgG Alexa Fluor 568 (Invitrogen, A11004), goat against rabbit IgG Alexa Fluor 568 (Invitrogen, A11011), and goat against mouse Alexa Fluor 488 (Invitrogen, A11001). Immunofluorescence was imaged using a Nikon A1-R confocal microscope. Cell proliferation was studied in histological sections of mouse heart, which were stained using an antibody against phosphorylated histone H3 (phosphorylated HH3) as described previously (Hinton et al., 2006). The proliferation index was calculated by determining the ratio of positively stained nuclei to the total number of nuclei in the hinge region of the valve. The phosphorylated Smad2/3 nuclei index was calculated in the same way. Images were analyzed using NIS-Elements software (Nikon). A total of four to six animals were studied per genotype per stage.

**Table 2.**
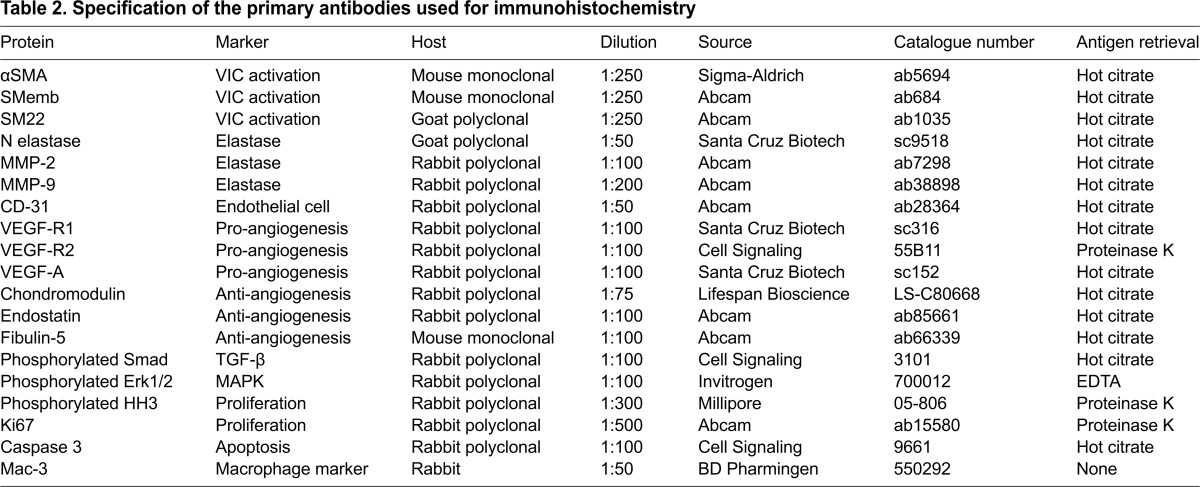
Specification of the primary antibodies used for immunohistochemistry

### Electron microscopy

Ultrastructure analysis was performed using a Hitachi 7600 transmission electron microscope (Hitachi Technologies). Aortic valve, ascending aorta and left ventricular myocardium were processed and analyzed as described previously (Hinton et al., 2010). In order to visualize collagen and elastic fibers, sections were counter stained with aqueous solutions of 5% tannic acid followed by 1% uranyl acetate, and then counterstained with lead citrate.

### QRT-PCR

Aortic valves were dissected from adult and aged mouse heart from *Emilin1*^−/−^ or *Emilin1*^+/+^ mice. Aortic valve tissue from three mice was pooled and RNA was isolated using the standard Trizol method. cDNA was generated using ~500 ng RNA followed by QRT-PCR and amplified by PCR using gene specific primers (supplementary material Table S1) (Hinton et al., 2010). Ct values were obtained using Bio-Rad software. The ΔΔCt method was used to represent the mRNA fold change. Three experiments were performed for each stage and genotype.

### Immunoblotting

Analyses were performed on aortic valves isolated from five stage- and genotype-matched mice, as described previously (Givvimani et al., 2012). A bicinchoninic acid protein assay kit (Thermo Scientific, Rockford, IL, USA) was used to estimate total protein, and 30 μg of protein lysate was loaded onto 8–12% SDS-PAGE gels and then transferred onto nitrocellulose membranes. The membranes were blocked with 3% non-fat dry milk in TBS Tween 20 and incubated with primary antibodies against MMP-2 (1:1000), MMP-9 (1:1000), phosphorylated Smad2/3 (1:300) and phosphorylated Erk1/2 (1:1000) overnight at 4°C. Immunoblots were probed with horseradish-peroxidase-conjugated secondary antibodies for 1 hour at room temperature and developed using chemiluminescence (Amersham Biosciences). After applying a stripping buffer (Thermo Fisher), the blots were re-probed with antibodies against GAPDH (Santa Cruz Biotechnology) or total Erk1/2 (Cell Signaling). Signal intensity was quantified using National Institutes of Health ImageJ software. The arbitrary pixel densities of each protein were normalized to GAPDH or total Erk1/2.

### Echocardiography

Cross-sectional, two-dimensional and color Doppler transthoracic echocardiography was used to assess cardiac structure and function *in vivo*. Mice were studied at the aged stage. Aortic valve structure and function were quantified using a mouse valve protocol that we developed in accordance with consensus guidelines (Cheitlin et al., 2003; Hinton et al., 2008; Baumgartner et al., 2009). A Visual Sonics Vivo 2100 Imaging System (Toronto) with a 30 MHz transducer was used. Mice were anesthetized with 1–2% iso-fluorane with continuous temperature and heart rate monitoring (Hinton et al., 2008; Hinton et al., 2010). The aortic root dimension was indexed by calculating the ratio of the aortic root to the ascending aorta as described previously (Kemna et al., 2009).

### Statistical analysis

Descriptive statistics were reported as the mean±s.e.m. Student’s *t*-test was used to compare groups using Graph Pad Prism software. A *P*-value of <0.05 was considered significant.

## Supplementary Material

Supplementary Material
