# Supplementary material for: TGF-β mediates early angiogenesis and latent fibrosis in an Emilin1-deficient mouse model of aortic valve disease
Source: Dis Model Mech. 2014 Aug;7(8):987–96. doi: 10.1242/dmm.015255 (PMC4107327; doi:10.1242/dmm.015255)
Supplement: Supplementary Material [file supp_7_8_987__index.html]

TGF-β mediates early angiogenesis and latent fibrosis in an Emilin1-deficient mouse model of aortic valve disease — Supplementary Material 

# TGF-β mediates early angiogenesis and latent fibrosis in an Emilin1-deficient mouse model of aortic valve disease

## DMM015255 Supplementary Material

**Files in this Data Supplement:**

- **Supplementary Material**
