# Supplementary material for: TGF-β mediates early angiogenesis and latent fibrosis in an Emilin1-deficient mouse model of aortic valve disease
Source: Dis Model Mech. 2014 Aug;7(8):987–96. doi: 10.1242/dmm.015255 (PMC4107327; doi:10.1242/dmm.015255)
Supplement: Supplementary Material [file supp_7.8.987_DMM015255.pdf]

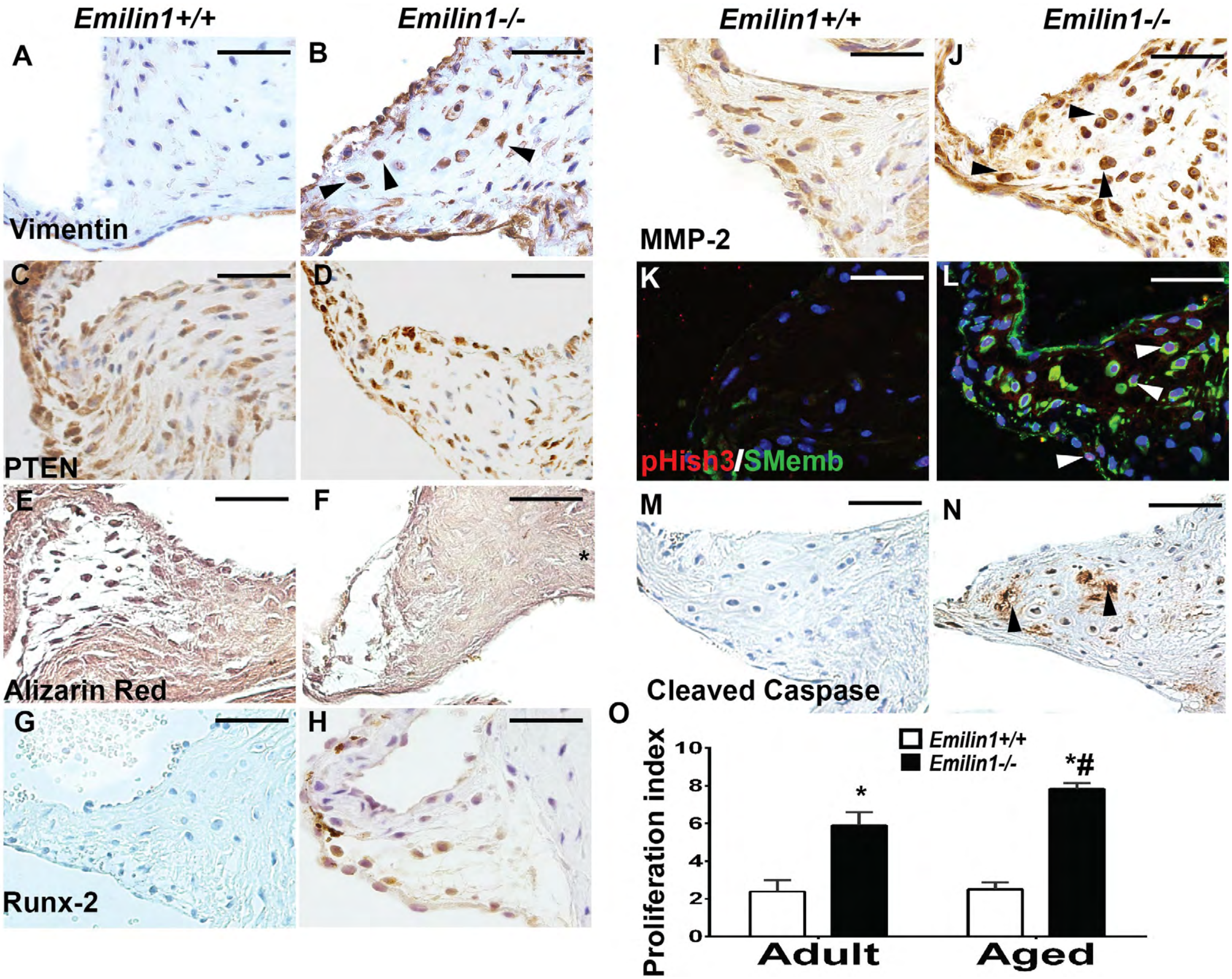

Supplementary Figure 1.

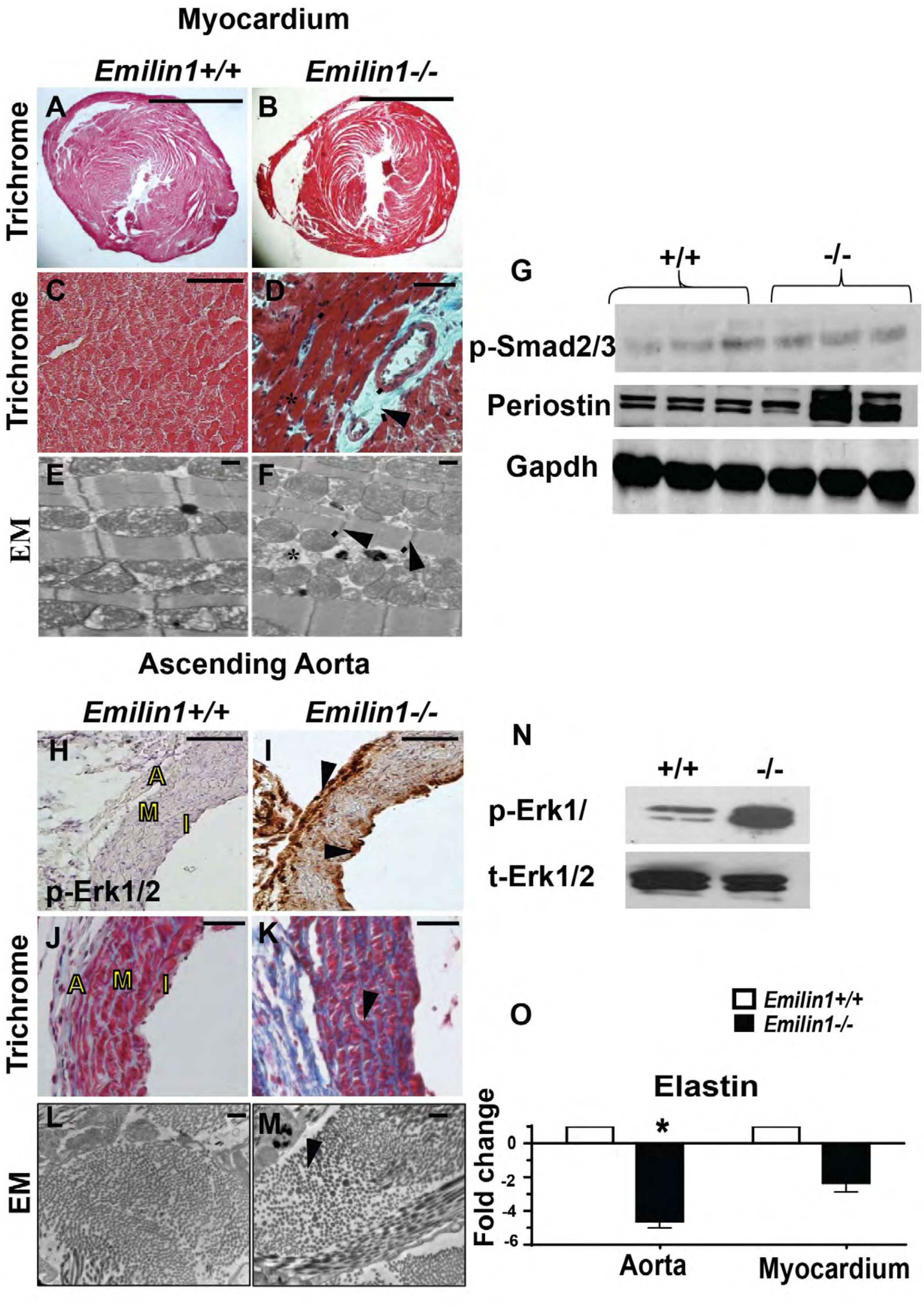

Supplementary Figure 2.

SUPPLEMENTARY FIGURE LEGENDS

Supplementary Fig S1. Photomicrograph showing altered expression of fibroblast marker vimentin in aged mutant aortic valves (arrowheads, B) when compared to age matched control (A). Panels C and D show unchanged PTEN expression in *Emilin1*$^{-/-}$ valves. *Emilin1*$^{-/-}$ valve did not show calcification at any stage as indicated by alizarin red (F) and runx-2 (H) staining. There was robust increase in expression of MMP-2 in aged *Emilin1*$^{-/-}$ mice (arrowheads, J) when compared to the control mice (I). Dual stained micrograph shows co-localization of p-HH3 (Red) and SMemb (Green) in *Emilin1*$^{-/-}$ mutant valves (arrowheads, L) when compared to control (K). Apoptosis assessment using Cleaved caspase-3 staining shows increased expression in aortic valve tissue of aged *Emilin1*$^{-/-}$ mice localized to the hinge region (arrowheads, N) when compared to *Emilin1*$^{+/+}$ mice. Panel O shows proliferative index analyzed using Ki67 staining. The scale bar (upper right corner each panel) is 500μm in A and N. *p<0.05 *Emilin1*$^{+/+}$ vs. *Emilin1*$^{-/-}$; $^{\#}p$<0.05 adult *Emilin1*$^{-/-}$vs. aged *Emilin1*$^{-/-}$.

Supplementary Fig S2. Trichrome stained short axis heart sections showing unaltered morphology and dimensions of left ventricular muscle in aged *Emilin1*$^{-/-}$ (B) vs. *Emilin1*$^{+/+}$ (A) heart. Photomicrograph showing collagen deposition in perivascular region of the myocardium in *Emilin1*$^{-/-}$(D), when compared to age matched *Emilin1*$^{+/+}$ (C) hearts. Ultrastructural analysis of left ventricular myocardium showing non-specific sarcomere abnormalities (arrows, F), as well as increased spaces between mitochondria and sarcomeres (star, F), in *Emilin1*$^{-/-}$ when compared to *Emilin1*$^{+/+}$ (E). Immunoblot showing p-Smad2/3 and periostin expression in the left ventricular myocardium of *Emilin1*$^{-/-}$ (G). Ascending aorta from aged *Emilin1*$^{-/-}$ mice demonstrating robust increase in p-Erk1/2 activation (I) and adventitial fibrosis (K) in comparison to age matched controls (H, J). Yellow color letters A, M and I correspond to adventitia, media and intima respectively. EM showing heterogeneity in collagen fiber size of ascending aorta from *Emilin1*$^{-/-}$ showing (arrows, M) when compared to *Emilin1*$^{+/+}$ (L) . Similarly, Immunoblot in panel (N) showing increased expression of p-Erk1/2 in ascending aorta from aged *Emilin1*$^{-/-}$ mice. mRNA expression of elastin is decreased in aged *Emilin1*$^{-/-}$ in aorta tissue (O) when compared to *Emilin1*$^{+/+}$ controls. The scale bar (upper right corner each panel) is 500μm in A and B; 50μm in C, D, H, I, J and K; 250nm in E, F, L and M. Mean ± SEM; * $p < 0.05$ aged *Emilin1*$^{+/+}$ vs. *Emilin1*$^{-/-}$.

**Supplementary Table 1: Specification of the Primers**			
Gene	Annealing Temp (^{0}C)		Primer Sequences
VEGF-A	55	F	CAC TGG ACC CTG GCT TTA CTG CT
		R	CTC ACC GCC TTG GCT TGT CAC
VEGF-R1	55	F	GAA ACC ACA GCA GGA AGA CG
		R	CTT TAT GCC CAG CAA GAT CG
VEGF-R2	55	F	GCC CTG CTG TGG TCT CAC TAC
		R	CAA AGC ATT GCC CAT TCG AT
Type I Collagen	55	F	ACC AGG AAT GCC TTG TTC TC
		R	CAT AAA GGG CCC ACT TGC TA
Type II Collagen	55	F	ACT GGT AAG TGG GGC AAG AC
		R	CCA CAC CAA ATT CCT GTT CA
Type III Collagen	60	F	AAG GCT GAA GGA AAC AGC AA
		R	TGG GGT TTC AGA GAG TTT GG
